# Launching a multi-level data strategy for rare eye diseases: a methodological case study from national to European scale

**DOI:** 10.1186/s13023-026-04505-0

**Published:** 2026-07-21

**Authors:** Camille Beluffi-Marin, Marilyne Oswald, Isabella Anna Vacchi, David Keegan, Bart Peter Leroy, Hélène Dollfus

**Affiliations:** 1 Laboratoire de Génétique Médicale, UMR_S INSERM U1112, IGMA (Institut de Génétique Médicale d’Alsace), Université de Strasbourg, Strasbourg, France; 2 FSMR SENSGENE Management Team, Hôpitaux Universitaires de Strasbourg, Strasbourg, France; 3 ERN-EYE Coordination Center, Hôpitaux Universitaires de Strasbourg, Strasbourg, France; 4 Department of Ophthalmology, Mater Misericordiae University Hospital, Dublin, Ireland; 5 University College Dublin (UCD), Dublin, Ireland; 6 Department of Ophthalmology, Ghent University Hospital, Ghent, Belgium; 7 Department of Head and Skin, Ghent University, Ghent, Belgium; 8 Center for Medical Genetics Ghent, Ghent University Hospital, Ghent, Belgium

**Keywords:** Rare eye diseases, Health data warehouse, Health care data secondary use, Interoperability, Data governance, SENSGENE, ERN-EYE, BNDMR, BaMaRa

## Abstract

Rare eye diseases bring unique challenges in clinical research and patient care due to their heterogeneity, low prevalence, and dispersed expertise. To address these challenges, an integrated multilevel data strategy has been developed in France and Europe, enabling structured, interoperable data collection and reuse across national and transnational initiatives. In this paper, we present a methodological framework using the metaphor of a spaceship to illustrate the vertical articulation of this ecosystem, from national infrastructures to European platforms. At the base of the spaceship lies BaMaRa, France’s national registry for rare diseases, which ensures the systematic collection of core clinical data at the point of care. The next stage is FREDD, a disease-specific data warehouse dedicated to rare eye conditions, developed by the SENSGENE network thanks to the France 2030 RaReTiA project. FREDD integrates granular phenotypic and genotypic data, allowing for in-depth research and cohort building. FREDD is fully interoperable with BaMaRa, ensuring data consistency and minimizing redundancies. The third level of the spaceship is REDgistry, the European registry for rare eye diseases coordinated by ERN-EYE. REDgistry allows for cross-border data harmonization and aligns with FAIR principles to facilitate data sharing and secondary use in international research. At the top of the structure is the European Health Data Space, a future platform enabling secure and standardized access to health data for research and policy-making at the EU level. At the core of this architecture remains the patient, whose data is the very foundation of the system. Although not always actively involved in data collection, the patient remains central to the purpose and structure of each data layer. Building and sustaining such a complex infrastructure requires the coordination of a wide range of stakeholders, including clinicians, research networks, data stewards, hospital IT departments, national authorities, and European institutions. The success of this multilevel model depends on shared standards, transparent governance, and sustained collaboration across all actors. Together, they form the propulsion system of the spaceship, enabling the structured reuse of high-quality data to accelerate research and improve care in the field of rare eye diseases.

## Background

### Heterogeneous development of rare disease data ecosystems across Europe and beyond

Rare diseases pose unique challenges to healthcare systems and researchers. Their low prevalence, clinical heterogeneity, and geographic dispersion make large-scale, robust studies based on databases difficult to conduct [1]. Nevertheless, high-quality, structured, and interoperable data collection is essential to advance scientific and medical knowledge, improve diagnosis and patient management, and develop new treatments [2]. However, the current landscape of rare disease data limits their reuse and integration for research and translational purposes.

Across Europe, rare disease data ecosystems have evolved through heterogeneous national strategies, often shaped by historical collaborations between clinicians, researchers, and specialized centers. Selected examples from Italy and the Netherlands highlight the diversity of national approaches within Europe. In Italy, the National Rare Diseases Registry (*Registro Nazionale Malattie Rare*, RNMR) established a federated framework connecting regional registries and national surveillance systems, coordinated by the Istituto Superiore di Sanità, with strong emphasis on standardized data collection and governance [3, 4]. This structure is complemented by initiatives such as the Italian Undiagnosed Rare Diseases Network (URDN), which reinforces the importance of multidisciplinary collaboration and cross-border data sharing for unresolved cases [5]. In the Netherlands, rare disease data collection relies on a more distributed model, structured around disease-specific registries hosted by university medical centers, with key contributions to FAIR data implementation through platforms such as MOLGENIS [6].

At the European level, several complementary initiatives have contributed to the development of interoperable and FAIR-oriented rare disease data ecosystems. These include data infrastructures such as RD-Connect [7] and Solve-RD [8], research coordination programs such as the European Joint Program on Rare Diseases [9] and ERDERA (European Rare Disease Research Alliance) [10], as well as the Joint Action JARDIN [11], which specifically aims to strengthen the integration of rare disease data at the European level. In parallel, the European Reference Networks (ERNs) [12], provide a clinical framework connecting expert centers across Member States to improve the diagnosis, management, and study of rare and complex diseases. Such expert networks have demonstrated the value of integrated approaches for cross-border research, diagnosis, and patient stratification [13].

Beyond Europe, the United States developed large-scale collaborative infrastructures such as the Rare Diseases Clinical Research Network (RDCRN) [14, 15] and the Undiagnosed Diseases Network (UDN) [16], which support nationwide collaboration in phenotyping, genomic diagnostics, and translational research.

In France, the last decades have seen the progressive structuring of rare disease care and data pathways through successive national programs *Plan National Maladies Rares* (PNMRs, National Rare Diseases Plan) [17–20]. This long-term strategy has supported the development of national infrastructures such as BaMaRa (*Base Maladies Rares*, Rare Disease database) and the BNDMR (*Banque National Des Maladies Rares*, National Rare Disease Bank) [21, 22], enabling national coordination for standardized data collection of rare disease information. Beyond their operational role, these infrastructures constitute the foundational layer upon which disease-specific databases, ERN registries, and future European data-sharing frameworks can progressively be connected.

### Rare eye diseases as a model for multi-layered data integration

Rare Eye Diseases (REDs) represent an archetypical field of rare diseases because of the number of Diseases (> 1000), the vast genetic heterogeneity, the unmet need for targeted therapies, the difficulties in performing trials due to the scattered population and the challenge in identifying patients eligible for clinical trials, consequently limiting the efficiency of the latter.

Based on the coordination of the National French network for rare sensory diseases SENSGENE [23], we have engaged in structuring and connecting RED data and gained first-hand insight into the extraordinary complexity of building a multi-tiered data architecture spanning from national infrastructures, including BaMaRa/BNDMR and the French Rare Eye Disease Database (FREDD) [24], to European-level systems such as REDgistry [25], the European registry of the ERN dedicated to eyes disease (ERN-EYE), and ultimately connecting to the broader European Health Data Space (EHDS) initiative [26]. This complexity arises not only from technical constraints but also from the need to align multiple operational and regulatory perspectives across the data lifecycle, involving clinicians responsible for data entry, hospital systems managing clinical workflows, registry teams ensuring data standardization, data protection officers overseeing regulatory compliance and governance bodies shaping implementation priorities and sustainability.

To conceptualize this complex and multi-layered structure, this paper proposes a metaphorical framework based on a spaceship, with patient data as its central passenger. The objective is to provide a structured lens through which to analyze the challenges of federated, multi-level rare disease data ecosystems. This paper presents a structured account of our experience, grounded in both a conceptual framework and the practical realities of implementing interconnected data infrastructures for rare diseases, illustrated here through the example of RED, with the challenges encountered and emerging solutions already providing valuable methodological insights into federated data ecosystem design.

## A patient-centered vision for research in rare diseases

The ultimate aim of rare disease research using databases is to translate data into improved care, faster diagnosis, and foster accessibility to clinical trials and ultimately treatments—wherever the patient is located at national level and in the EU. This goal requires a unified vision across multiple actors and levels as well as swift and easy to use tools.

From the moment when a patient enters the healthcare system, his/her data may be captured and stored in different silos—hospital records (paper or electronic), national registries, disease-specific cohorts, or European platforms. Each system operates under distinct purposes, ranging from routine clinical care and care coordination to structured data collection for research and secondary use. As a result, each system is governed by its own operational rules and legal frameworks, reflecting differences in data ownership, consent models, and regulatory requirements.

From the patient’s view, such complex, multi-layered data collection structures should remain invisible whereas an easy conduit should enable meaningful patient-centered research through facilitated access to data. This is especially critical in rare diseases, where data scarcity makes every contribution valuable and where transnational research is often the only way to reach statistically meaningful insights. Recent studies have shown that patients generally support the secondary use of their health data for research purposes, provided that such use is transparent, secure, and directed towards the public good [27, 28]. Concerns about data misuse, loss of privacy, and commercialization without benefit sharing remain prevalent [29]. In the context of rare diseases, a large portion of patients tend to express a particularly strong willingness to contribute to data sharing initiatives, reflecting an awareness of the need to collectively aggregate fragmented data across borders to advance research and care [30, 31]. Ensuring that data infrastructure serves the patient-centered vision, while maintaining trust through robust governance and clear communication, is thus essential for the success of federated data collection to support meaningful research efforts for rare diseases.

## The spaceship metaphor: an architecture of interconnected data floors

To help conceptualize this challenge at the French level, taken as an example herein, we propose the metaphor of a spaceship program. From this perspective, patient data are not passive data points but rather travelers on a mission. Just like a spaceship composed of modular floors, each with its own technical and operational logic, rare disease data infrastructures require harmonized coordination without compromising the autonomy of each layer. The strength of the system lies not in centralization, but in its interoperable orchestration.

**Fig. 1:**
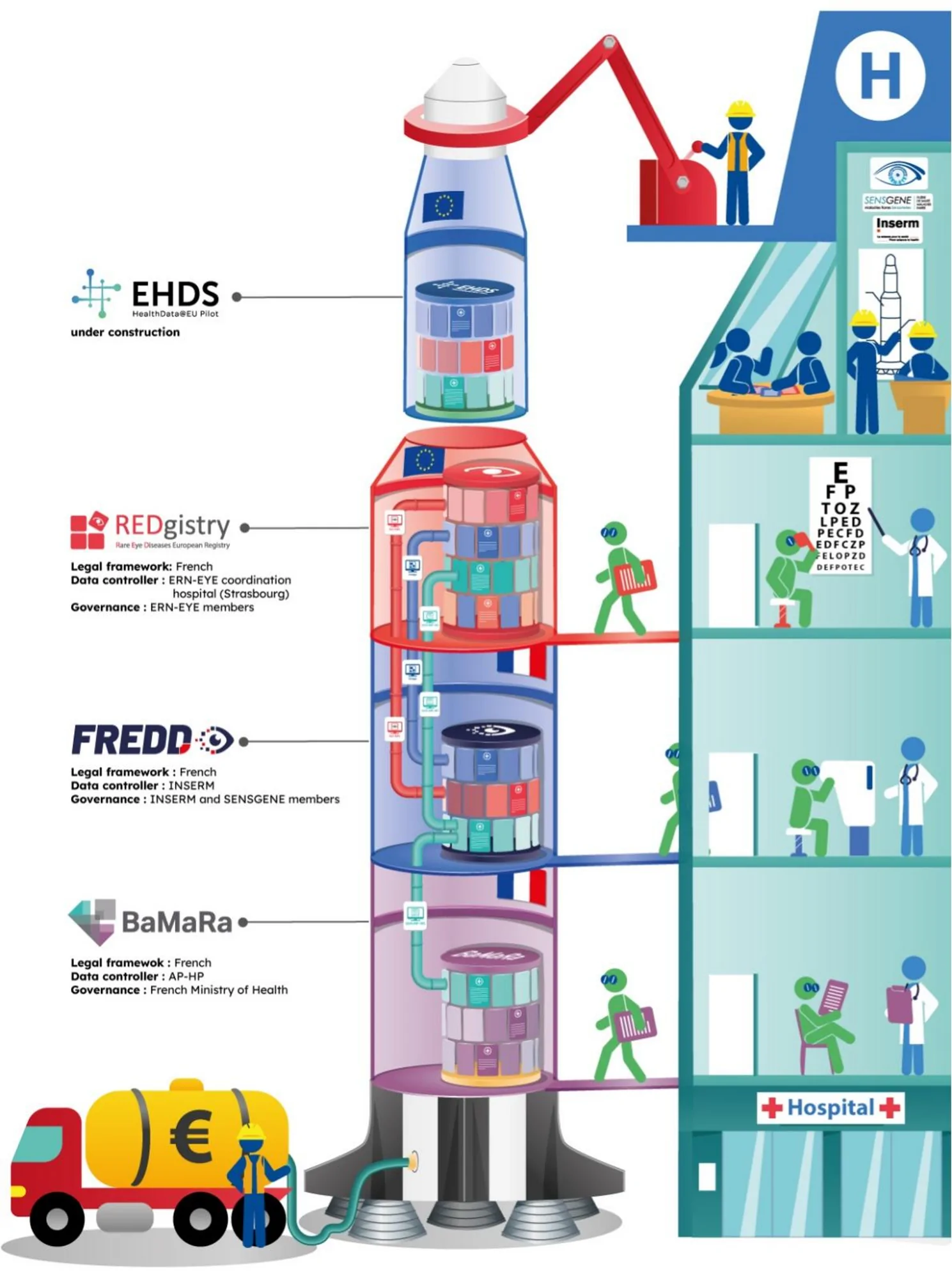
Conceptual spaceship metaphor representing the multi-layered rare disease data ecosystem. Each floor illustrates a distinct database environment — BaMaRa/BNDMR at the national level, FREDD as a disease-specific national registry, REDgistry as the ERN-EYE European registry, and the EHDS as the future European secondary use framework. The figure also depicts data circulation between infrastructures, interoperability links across national and European levels, and the corresponding data controllers associated with each database environment

Within this conceptual framework, illustrated in Fig. 1, each floor of the spaceship represents a distinct level of data infrastructure. This layered architecture illustrates the French RED ecosystem, which constitutes a particularly integrated vertical system linking a nationwide registry (BaMaRa/BNDMR), a disease-specific research platform (FREDD), and a European ERN registry infrastructure (REDgistry), all connected through a continuous interoperability framework across national and European levels.

### First floor: BaMaRa, the national foundation

At the base of the structure lies the BNDMR, the French national registry for rare diseases. Launched in 2019 during the second PNMR and managed by the *Assistance Publique–Hôpitaux de Paris* (AP-HP) as data controller [32], it has been developed under the legal obligations of the French health system. The BNDMR is based on a centralized architecture with the BaMaRa software supporting collection of structured data on rare disease patients from hospitals across France. The BaMaRa dataset was built around the Rare Disease Minimum Data Set (SDM-MR), which defines the mandatory minimum information to be collected for each patient diagnosed with a rare disease. It forms the structural foundation of the spaceship—essential for stability and alignment.

### Second floor: FREDD, the disease-specific enrichment layer

Built above the BaMaRa/BNDMR floor is FREDD, a disease-specific registry dedicated to RED. FREDD has been developed and supported by SENSGENE, and its creation was made possible through the PNMR3 and the *France 2030* initiative [33], specifically by answering the call for projects “*Accelerating Research and Innovation on Rare Diseases through Databases*.” RaReTiA (ANR-21-PMRB-0009) was one of 11 eleven selected rare diseases projects. It is a pioneering initiative designed to establish FREDD and leverage its data for groundbreaking research. FREDD’s data controller is the *Institut National de la Santé et de la Recherche Médicale* (INSERM) [34], which ensures compliance with French regulatory frameworks and ethical principles. The project also sought to enhance FREDD’s information system in collaboration with *France Cohortes* [35], an infrastructure playing a pivotal role as a service provider, ensuring the robustness, security and interoperability of FREDD’s information system. FREDD’s dataset partially overlaps with BaMaRa, from which it imports core clinical data, including the SDM-MR. To facilitate data reuse and avoid duplicate manual entry, the FREDD team developed FREDDEX [36], an interoperability tool that maps and transforms BaMaRa data into the FREDD data model. FREDD is subsequently enriched with genetic results and detailed ophthalmological information, including imaging and visual function tests, which are critical for rare eye disease research. The database has been authorized by French regulations in 2024 [36] and the data collection started in 2025. As a disease-specific research infrastructure, it complements the national rare disease ecosystem by collecting detailed ophthalmological and genetic data while supporting interoperability with European initiatives.

### Third floor: REDgistry, the European integration layer

The third floor of the spaceship is REDgistry, the central registry of ERN-EYE, initiated in 2022. Following initially a centralized model, it aims to collect data from all ERN-EYE Healthcare Providers (HCPs) across Europe. Although data governance remains distributed among participating centers, legal responsibility currently lies with France. Structurally, the REDgistry dataset closely mirrors that of FREDD, as both integrate the SDM-MR, from which the EU Joint Research Centre (JRC) minimum data set was developed. To facilitate interoperability between national and European infrastructures, a dedicated transfer module inspired by FREDDEX is currently under development to support the automated transformation and integration of FREDD data into REDgistry. REDgistry is designed to serve as a core repository for future topic- or disease-specific satellite registries, such as RETgistry, which will focus on the collection of retinal imaging and functional data. Together, these infrastructures illustrate a key principle of the ERNs: enabling cross-border collaboration through harmonized yet adaptable data models. The scientific value of such interoperable ecosystems has been demonstrated by initiatives such as Solve-RD, which demonstrated that collaborative genomic reanalysis across European rare disease networks could yield substantial numbers of new diagnoses through harmonized data sharing, expert review, and cross-border integration of clinical and genomic datasets [37].

### Fourth floor: the EHDS, the interstellar gateway

At the top of the architecture lies the European Health Data Space (EHDS) [38], a major initiative of the European Commission designed to establish a common legal, technical, and governance framework for the secure exchange and secondary use of health data across Europe. One of its main objectives is to facilitate the discovery, accessibility, and reuse of health data for research, innovation, public health, and policy-making purposes across Member States. To achieve this objective, the EHDS will rely on the HealthData@EU infrastructure [39], which is intended to enable researchers to identify and access relevant datasets across distributed national and European data ecosystems. Following the adoption of Regulation (EU) 2025/327 [40] and its entry into force in March 2025, the EHDS represents the final stage of the journey: the integration of structured, interoperable, and securely governed health data into a European ecosystem capable of supporting large-scale collaborative research. This floor is the launchpad, opening the door to continent-wide collaboration and discovery.

## The patient journey and consent across the data floors

As the patient’s data progress through the different layers of the rare disease data infrastructure, the rules governing consent and data usage vary from one floor to the other. Some systems are based on opt-in consent, requiring active agreement before data can be shared, while others operate under an opt-out model, where data are included by default, unless the patient explicitly objects.

For example, both the BNDMR and FREDD operate under an opt-out legal framework that seeks to balance large-scale data collection with the protection of individual rights. Patients are informed about the purposes of data collection and reuse through dedicated information materials and transparency portals, and may exercise their GDPR (General Data Protection Regulation) rights, including access, rectification, objection, restriction of processing, and, where applicable, erasure [41].

At the European level, governance models are more heterogeneous. Several ERN registries, including REDgistry and RETgistry, rely on explicit patient consent for participation and data sharing across borders.

At the European level, the EHDS aims to enable the secondary use of health data for research and public health purposes under a regulated access framework based on an opt-out model for patient participation^1^ [38]. While this right is harmonized across Member States, national authorities will remain responsible for implementing the operational mechanisms governing data access, authorization procedures, and oversight, which may result in some variation in practice.

In principle, this multi-layered landscape should allow patients to remain informed and empowered regarding how their data are used across systems, maintaining a strong degree of control over personal data that derive directly from their care and are subject to fundamental rights of protection and autonomy. In practice, however, the coexistence of heterogeneous consent models, regulatory frameworks, and data infrastructures across all layers makes this continuity difficult to achieve. This tension between a patient-centered ideal of empowerment and a fragmented operational reality highlights the need for more coherent and interoperable governance mechanisms that can sustain both scientific progress and patient trust.

## The control center : coordinating a distributed architecture

### Governance of a distributed architecture

Coordinating distributed rare disease data infrastructures requires coherence across multiple legal, organizational, and technical environments that extend beyond traditional governance boundaries. In any complex structure such as a metaphorical spaceship, each layer of the architecture operates under its own data controller, governance bodies, and regulatory obligations, while remaining compliant with the GDPR and applicable national legislation. Across infrastructures, governance generally relies on a combination of strategic oversight and scientific and ethical review. Steering committees guide the evolution of the infrastructure, while scientific and ethical committees oversee methodological quality, data access policies, and compliance with ethical requirements. However, formal governance structures alone are not sufficient to ensure sustained alignment across levels of implementation, and additional operational mechanisms are required to prevent fragmentation between national and European infrastructures.

In practice, such alignment is rarely achieved through abstract coordination alone but depends on key integrative actors embedded within the ecosystem. In the RED ecosystem, this role is fulfilled by the close and structured collaboration between the SENSGENE network and the ERN-EYE coordination framework. Their integrated functioning enables sustained alignment of data models, governance practices, and data collection strategies across national and European levels, thereby providing both stability and adaptability within a complex regulatory environment.

This integrative function is reflected in the FREDD governance, which includes representatives from the BNDMR ecosystem to ensure consistency between the national registry layer (BaMaRa/BNDMR) and the disease-specific research environment. At the European level, REDgistry governance is organized at consortium level, reflecting the distributed nature of ERN-EYE, with coordination mechanisms designed to harmonize data capture, ensure interoperability of core variables, and support cross-border aggregation of rare eye disease cohorts.

Looking forward, the EHDS is expected to introduce an additional coordination layer for the secondary use of health data across Europe. Rather than replacing existing governance structures, it aims to connect national and European infrastructures through common frameworks for interoperability and data access, supported by a governance model involving national Health Data Access Bodies (HDABs), digital health authorities, and a European coordination board responsible for aligning implementation across Member States. While access decisions remain primarily at national level through HDABs, this structure establishes a shared European framework for authorization procedures, oversight, and cross-border data discovery, while preserving the respective responsibilities of data holders and national authorities.

### Resource optimization and sustainability

Beyond governance and architectural considerations, this multi-level framework also provides a pragmatic approach to addressing the persistent challenges of fragmented funding and human resource turnover. By co-localizing the national (SENSGENE) and European (ERN-EYE) management teams in the same physical and organizational space, the strategy enables a highly efficient mutualization of expertise. Specialized profiles, such as data stewards and project managers, can bridge both national and transnational tasks, thereby reducing the need for redundant recruitments and optimizing limited financial resources. Furthermore, the technical verticality of the architecture drastically minimizes operational burdens at the clinical level: because core clinical data is automatically inherited from BaMaRa to FREDD via a FREDDEX (and, in the future, extended to the FREDD–REDgistry transfer layer), double-data entry is limited for hospital staff. This interoperability by design acts as a significant resource-saving mechanism, allowing scattered clinical teams to contribute to high-level research without increasing their workload. Ultimately, rather than waiting for a hypothetical stabilization of long-term public funding, this multi-level strategy demonstrates that structural interoperability and centralized network coordination are, in themselves, powerful tools to overcome human and financial constraints.

In this context, the sustainability of the entire structure does not rely solely on infrastructure, but also emerges from the coherence and interdependence of the different components of the system. The tight integration between governance structures, operational mechanisms, and collaborative networks across national and European levels creates an architecture in which each layer reinforces the others, thereby contributing to the overall resilience of the ecosystem.

## Conclusion

### Launching into the rare disease data galaxy: the need for resources to launch

Building this multi-level, interoperable and ethically robust spaceship for rare disease data is not just a technical or regulatory challenge — it is also a financial and human resource challenge. Each floor, each connector and each governance structure require sustainable investment to be fully operational, secure and scientifically valuable. A major hurdle remains the recruitment and retention of skilled personnel able to manage the complexity of such infrastructures, as these projects require highly specialized profiles that are difficult to attract and sustain over time. Without long-term staffing strategies, the loss of expertise triggers repeated recruitment cycles, undermining project continuity and adaptation to evolving challenges.

Despite the clear public health and research benefits, funding for such infrastructures often remains fragmented, short-term, or dependent on project-based grants. This threatens not only their long-term stability, but also their ability to evolve with emerging technologies, integrate new data sources, or respond quickly to regulatory changes. To ensure the sustainability of both the infrastructure and the expertise required to run it, a more comprehensive, visionary approach to long-term funding and human resource management is essential.

To ensure that the spaceship can take off and navigate long-term missions, coordinated funding mechanisms must be developed at national and European levels. These should support both the technical backbone—servers, information system, software for data processing—as well as the human expertise needed for project management, data curation and legal compliance.

Moreover, funding must reflect the strategic importance of data as a health system infrastructure, not just as a research tool. Investing in rare disease data ecosystems is investing in precision medicine, equity of access, and innovation.

Beyond the sustainability of individual projects, maintaining Europe’s competitiveness in rare disease research will require a long-term and coordinated investment strategy. The implementation of interoperable data ecosystems, the EHDS, and future AI-driven research infrastructures cannot rely solely on short-term project funding. The European Commission and Member States must recognize health data infrastructures as strategic assets and commit to sustained financial support for their development, maintenance, and evolution. Equally important, academic institutions, hospitals, and research organizations must invest in stable career pathways for specialized professionals—including data stewards, project managers, data scientists, interoperability experts, and regulatory specialists—whose expertise is essential for transforming fragmented health data into actionable scientific knowledge. Without such structural investments in both infrastructure and human capital, Europe risks limiting its capacity to fully exploit the scientific, clinical, and economic opportunities offered by health data at scale.

Without this sustained support, even the most ambitious and well-designed spaceship may never be seen to take off.

## Data Availability

Not applicable.

## Footnotes

1 Article 71 = “Right to opt out from the processing of personal electronic health data for secondary use”.

## Abbreviations

AP-HP: Assistance publique Hôpitaux de Paris
BaMaRa: Base maladies rares
BNDMR: Banque nationale de données maladies rares
EHDS: European health data space
ERDERA: European rare disease research alliance
ERICA: European rare disease research coordination and support action
ERN: European reference network
FREDD: French rare eye disease database
HCP: Health care provider
INSERM: Institut National de la Santé et de la Recherche Médicale
JRC: Joint research center
IT: Information technology
PNMR: Rare diseases national plan
RED: Rare eye disease
SDM-MR: Rare disease minimum data set
